# Evidence-based decision support for pediatric rheumatology reduces diagnostic errors

**DOI:** 10.1186/s12969-016-0127-z

**Published:** 2016-12-13

**Authors:** Michael M. Segal, Balu Athreya, Mary Beth F. Son, Irit Tirosh, Jonathan S. Hausmann, Elizabeth Y. N. Ang, David Zurakowski, Lynn K. Feldman, Robert P. Sundel

**Affiliations:** 1 SimulConsult, Chestnut Hill, MA USA; 2 DuPont Hospital for Children, Wilmington, DE and Thomas Jefferson University, Philadelphia, PA USA; 3 Boston Children’s Hospital and Harvard Medical School, 300 Longwood Avenue, Boston, MA 02115 USA; 4 Edmond and Lily Safra Children’s Hospital, Tel-Hashomer, Ramat-Gan, Israel and Tel Aviv University, Tel Aviv, Israel; 5 National Univesity Hospital, Singapore, Singapore

**Keywords:** Pediatric rheumatology, Diagnosis, Diagnostic errors, Decision support, Computer software, Medical informatics

## Abstract

**Background:**

The number of trained specialists world-wide is insufficient to serve all children with pediatric rheumatologic disorders, even in the countries with robust medical resources. We evaluated the potential of diagnostic decision support software (DDSS) to alleviate this shortage by assessing the ability of such software to improve the diagnostic accuracy of non-specialists.

**Methods:**

Using vignettes of actual clinical cases, clinician testers generated a differential diagnosis before and after using diagnostic decision support software. The evaluation used the SimulConsult® DDSS tool, based on Bayesian pattern matching with temporal onset of each finding in each disease. The tool covered 5405 diseases (averaging 22 findings per disease). Rheumatology content in the database was developed using both primary references and textbooks. The frequency, timing, age of onset and age of disappearance of findings, as well as their incidence, treatability, and heritability were taken into account in order to guide diagnostic decision making. These capabilities allowed key information such as pertinent negatives and evolution over time to be used in the computations. Efficacy was measured by comparing whether the correct condition was included in the differential diagnosis generated by clinicians before using the software (“unaided”), versus after use of the DDSS (“aided”).

**Results:**

The 26 clinicians demonstrated a significant reduction in diagnostic errors following introduction of the software, from 28% errors while unaided to 15% using decision support (*p* < 0.0001). Improvement was greatest for emergency medicine physicians (*p* = 0.013) and clinicians in practice for less than 10 years (*p* = 0.012). This error reduction occurred despite the fact that testers employed an “open book” approach to generate their initial lists of potential diagnoses, spending an average of 8.6 min using printed and electronic sources of medical information before using the diagnostic software.

**Conclusions:**

These findings suggest that decision support can reduce diagnostic errors and improve use of relevant information by generalists. Such assistance could potentially help relieve the shortage of experts in pediatric rheumatology and similarly underserved specialties by improving generalists’ ability to evaluate and diagnose patients presenting with musculoskeletal complaints.

**Trial registration:**

ClinicalTrials.gov ID: NCT02205086

**Electronic supplementary material:**

The online version of this article (doi:10.1186/s12969-016-0127-z) contains supplementary material, which is available to authorized users.

## Background

Children with rheumatologic diseases face a serious shortage of trained specialists. As of the end of 2015, 325 pediatric rheumatologists in the United States had active board certification [1], well below even the most conservative estimates of an optimal rheumatologic work force [2]. The lack of access to pediatric rheumatology care is exacerbated by the fact that many of these specialists are not full-time clinicians. Further, pediatric rheumatologists generally work at academic medical centers in large urban areas; [3] as of 2015, 22 states had two or fewer pediatric rheumatologists and eight had none at all. This results in severely restricted geographical access to pediatric rheumatologists, with an earlier study finding that 24% of children in the United States lived more than 80 miles from a pediatric rheumatologist [4].

The lack of subspecialty availability and its negative impact on patient care are not limited to pediatric rheumatology, nor is it unique to the United States. A white paper by the World Forum on Rheumatic and Musculoskeletal Diseases found that of 26 countries for which data were available, only France, Uruguay, Australia and the US had more than one rheumatologist per 100,000 people, many of whom had administrative or research responsibilities that further limited their availability to patients [5]. In sub-Saharan Africa, fewer than 20 rheumatologists are available to serve more than 800,000,000 people [6]. The shortage has been particularly acute for children, with an inadequate number of pediatric rheumatologists on all continents; as of 2011, only two pediatric rheumatologists served all of Africa [7]. For many patients with rheumatologic disorders, the only option is to be treated by primary care providers, many of whom are untrained in rheumatology [8].

Features of rheumatologic conditions may exacerbate the lack of access to pediatric specialists. Signs and symptoms are often not specific, resulting in gatekeepers having difficulty identifying which patients to refer to pediatric rheumatologists. The most common reasons for pediatric rheumatology referrals are joint pain or swelling, abnormal results on lab tests such as erythrocyte sedimentation rate (ESR) and anti-nuclear antibody (ANA), and unexplained fevers, but these are often caused by infectious, genetic or orthopedic conditions [9]. The result is that conditions more appropriately diagnosed and managed by other caregivers occupy appointments that would otherwise be available for children with actual rheumatologic conditions. Similarly, inadequate numbers of specialty clinics within a reasonable distance often force generalists to choose between a closer or earlier evaluation by an adult rheumatologist or non-rheumatology pediatric specialist and a later, less accessible referral to a fully trained pediatric rheumatologist [10]. Such delays in receiving optimal care from a qualified expert often result in demonstrably worse outcomes [11, 12], particularly in rapidly progressive conditions such as Kawasaki disease [13].

Despite the fact that the inadequate number of pediatric rheumatologists has been recognized for more than a decade, various attempts to ameliorate the shortage have been ineffective. In 2007, a total of 81 trainees were enrolled in United States Pediatric Rheumatology three-year sub-specialty training programs; in 2015 there were 85 [1]. These numbers include foreign medical graduates who plan to return to their native countries to practice, yet even the total average number of fellows is barely keeping pace with the rate at which pediatric rheumatologists leave the work force due to retirement and decreasing clinical responsibilities [14]. Alternative approaches for increasing the availability of rheumatologist care for children, such as hiring nurse practitioners and other physician extenders [15] or use of virtual consultations using telemedicine [16] either have had minimal impact or are of unproven quality.

Such concerns about the inadequacy of the pediatric rheumatology workforce, mounting costs of diagnosis [17] as well as the potential for diagnostic errors in complex subspecialties like rheumatology [18], have combined with rapid technological advances in data processing, electronic health records [19] and genome-phenome analysis [20] to rekindle interest in computer assisted diagnosis. Ready access to diagnostic decision software that allows generalists and specialists to minimize referral and diagnostic problems would be an important advance in patient care and resource utilization. Evaluating such decision support tools, however, involves a key challenge. Assessments are most easily done using common diseases, for which many cases can be found, but clinicians value decision support most when an unusual or unfamiliar condition is being considered. To meet this challenge, we and others have used case vignettes of real patients and studied responses before and after introduction of decision support tools [21–23].

In this report we describe a decision support tool, SimulConsult®, which uses a statistical pattern-matching approach to inform diagnostic assessments. Its viewable database consists of evidence-based information derived from the medical literature and curated by experts. As the first test of the usefulness of this diagnostic tool we assessed its accuracy in supporting diagnosis and workup by pediatric rheumatologists and non-pediatric rheumatologists.

## Methods

The SimulConsult® diagnostic decision support tool is based on Bayesian pattern matching taking into account the temporal pattern of each finding in each disease [23]. The SimulConsult® tool was first used to support the diagnosis of genetic and neurologic conditions. Coverage of rheumatologic diseases was then added to the existing version of the software in a “curation” process in which disease findings were recorded. For each disease, the decision support software takes into account the prevalence at different ages, variations between genders, treatability, and family history. For each finding in each disease, frequency, age of onset, and age of disappearance (if applicable) are also incorporated. This quantitation and granularity of information increases the ability to use data in the diagnostic process, including pertinent negative results, efficacy of treatment and temporal evolution of diseases. In addition, this information forms the basis for iterative diagnosis, whereby suggestions of other clinical or laboratory findings likely to be therapeutically useful and cost effective in distinguishing between diagnoses are offered by the software [24]. Suggestions offered by the software are prioritized according to disease severity and actuity, the urgency to make a diagnosis and the potential benefits of treatment. The software is available to medical professionals and their trainees for free after a simple registration at www.simulconsult.com.

The curated information was entered by rheumatology fellows and junior faculty members using textbook and literature resources, and then edited by senior rheumatologists. Curation of findings (signs, symptoms and lab values) in rheumatologic conditions was reviewed for completeness using a checklist of 46 core clinical rheumatology findings developed as part of this effort. At the time of the current study, the tool covered 5405 diseases including the previously curated neurologic and genetic content as well as the newly added rheumatologic diseases. An average of 22 findings per disease are described.

Twenty-six testers were asked to evaluate eight case vignettes of real patients with confirmed diagnoses (six had pediatric rheumatologic diagnoses; two had other conditions with some rheumatologic findings). The cases were synopsized in vignettes by a senior pediatric rheumatologist (RPS) and averaged 295 words. Of the 26 testers, 13 were “junior” clinicians in the final year of training or first year after completing specialty or sub-specialty training. The remaining 13 had been practicing for at least 10 years (“senior”) (Table 1). Ten of the 26 testers were pediatric rheumatologists, nine were pediatric emergency medicine physicians and seven were general pediatricians. Of the 26 testers, 12 were male and 14 were female. All testers completed all cases. None of those who curated information or reviewed it served as testers.

**Table 1:** Mix of testers

	General Pediatrics	Emergency Medicine	Pediatric Rheumatology	Total
Junior	3	5	5	13
Senior	4	4	5	13
TOTAL	7	9	10	26

After reading each vignette and before using the decision support software, testers could use other resources such as books, papers and general web searches. After using such resources, but before using the diagnostic software, testers generated ranked “Baseline” lists of likely diagnoses and preferred laboratory tests, imaging studies, and consultations from lists of generally available resources. Each tester then used the decision support software to reassess each vignette, and again provided diagnosis and workup lists (“Intervention”). Building on our earlier study [23], testers were allowed to select from lists with specific diagnoses (e.g., juvenile psoriatic arthritis) or tests (e.g., magnetic resonance angiogram) as well as categories of diseases (e.g., arthritis) or bundles of tests with multiple possible findings (imaging study). After finishing each case, testers completed a questionnaire about the time used for research and for entering findings. On the first and last case, they also answered several open-ended questions about the process. Identities of all testers were blinded from curators who scored responses.

Information entered into the software was recorded as present or absent, and if known, timing or onset, allowing analysis of whether tester diagnostic errors resulted from user-input errors.

In order to assess subjects’ performances, rank-ordered lists of differential diagnoses and recommended diagnostic studies were prepared by the expert who had previously been involved in each case. These lists served as the “gold standard” against which tester responses were evaluated. All gold standard differential diagnoses included the proven diagnosis as #1. Two key quality measures of the diagnostic lists generated by study subjects were calculated: errors (tester’s differential diagnosis lists that omitted the correct diagnosis) and relevance (fraction of diagnoses in the tester’s differential diagnosis that were also in the gold standard). Corresponding measures were also calculated for workup.

The evaluation and analysis of making a diagnostic error (the binary outcome in the study) from a statistical perspective included generalized estimating equations or a GEE approach to account for the eight case vignettes confronted by each of the 26 testers before and after use of the DDSS software tool in decision making. The statistical analysis included the within-subject correlation (since these are not independent) and used a binomial distribution and a logistic regression model because an error in diagnosis is a binary (yes or no) outcome [25]. The Wald test distributed as a chi-squared statistic was used to determine whether the software tool aided in the reduction of diagnostic errors as well as to compare the outcome-based diagnostic performance according to level of training and particular medical specialty. Two-tailed *p* < 0.05 was considered statistically significant. IBM/SPSS software version 21.0 was used for analysis of the data (IBM Corporation, Armonk, NY).

## Results

### Effect on diagnostic errors

**Fig. 1:**
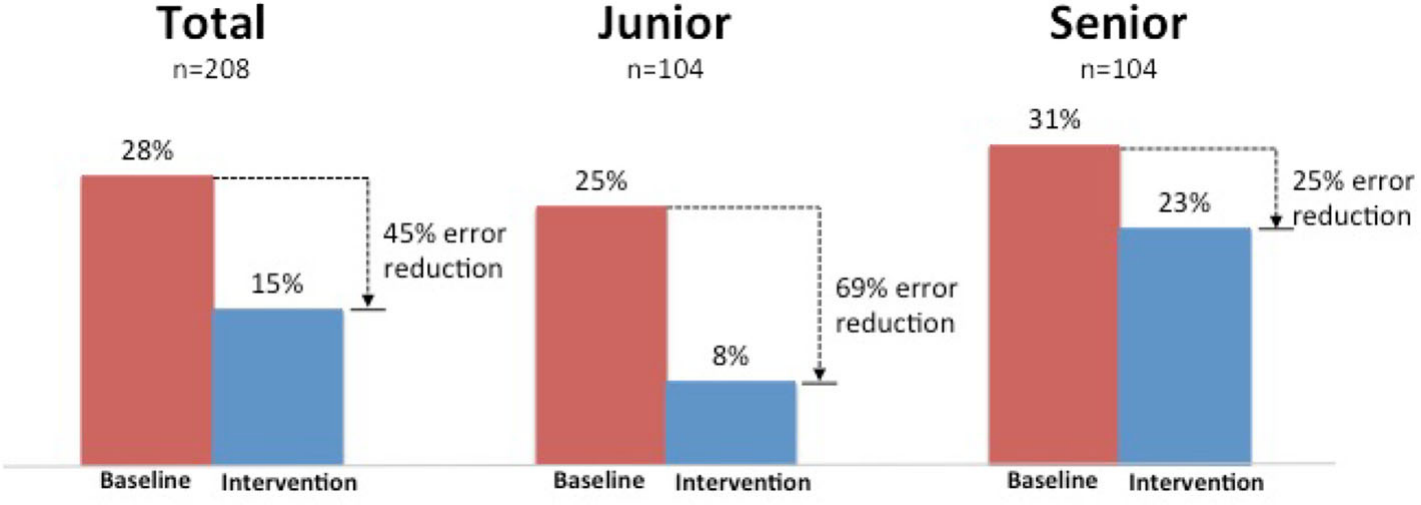
Diagnostic errors by seniority (All 26 testers, 8 cases each = 208 testing instances)

The case vignettes were difficult enough that testers did not include the correct diagnosis, or even its category, in the differential diagnosis (“diagnostic error”) in 28% of case testing instances before using the decision support (“baseline”). This was despite the fact that they were allowed to use other resources such as textbooks, articles or Web searches. Results of all eight cases together indicate a significant overall effect of the software in reducing diagnostic errors from an average of 28 to 15% overall, a relative decrease of 45% (Fig. 1; Wald test = 19.88, *p* < 0.0001). Junior clinicians benefitted more than did senior clinicians (Wald test = 6.26, *p* = 0.012). There was no significant difference between male and female clinicians (Wald test = 1.07, *p* = 0.30).

**Fig. 2:**
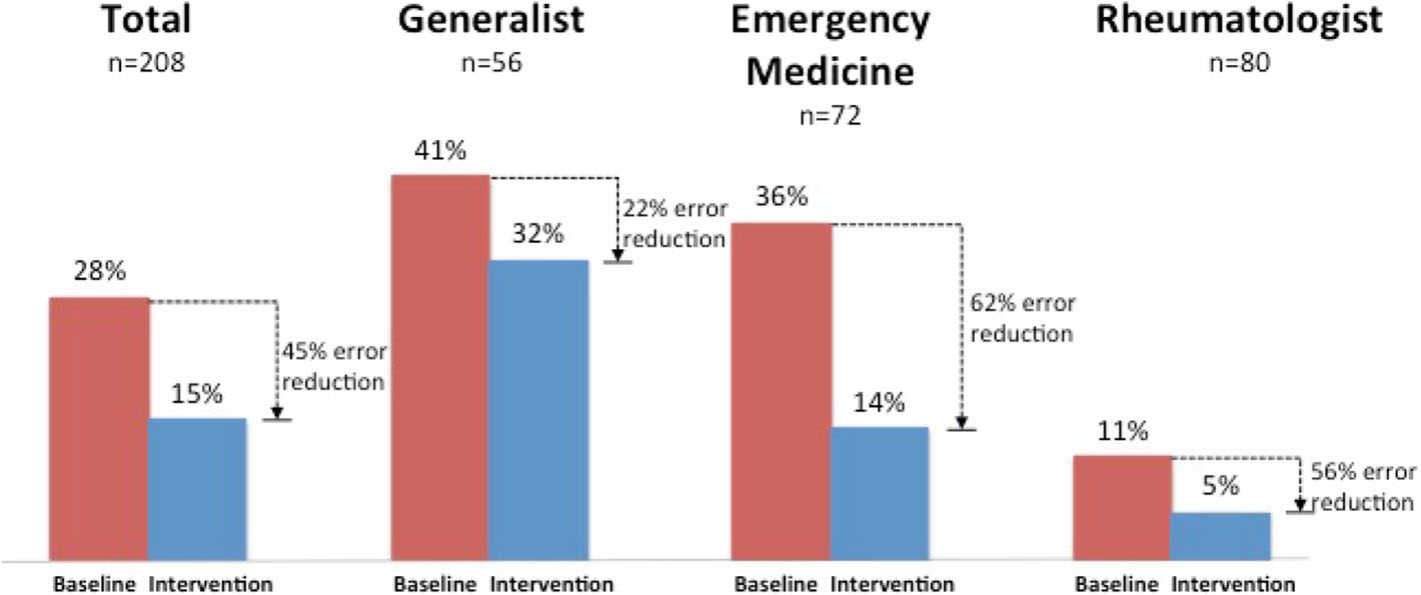
Diagnostic errors by specialty (208 discrete tests)

Error reduction was significantly larger for emergency medicine physicians as compared to generalists and rheumatologists (Fig. 2; Wald test = 6.21, *p* = 0.013).

The number of conditions listed in the differential diagnosis declined both when the tester’s diagnosis was supported by the decision support (“unchanged correct”, as assessed from the instances in which there was no diagnostic error either at baseline or after the intervention) as well as in cases where the response shifted from diagnostic error to no error (“reduced error”) (Table 2).

**Table 2:** Number of diseases in differential diagnosis: mean (median)

	All (*n* = 176)	Unchanged correct (*n* = 143)	Reduced error (*n* = 33)
Baseline	4.6 (4)	4.4 (4)	5.2 (5)
Post-intervention	3.5 (3)	3.4 (3)	4.2 (4)

**Fig. 3:**
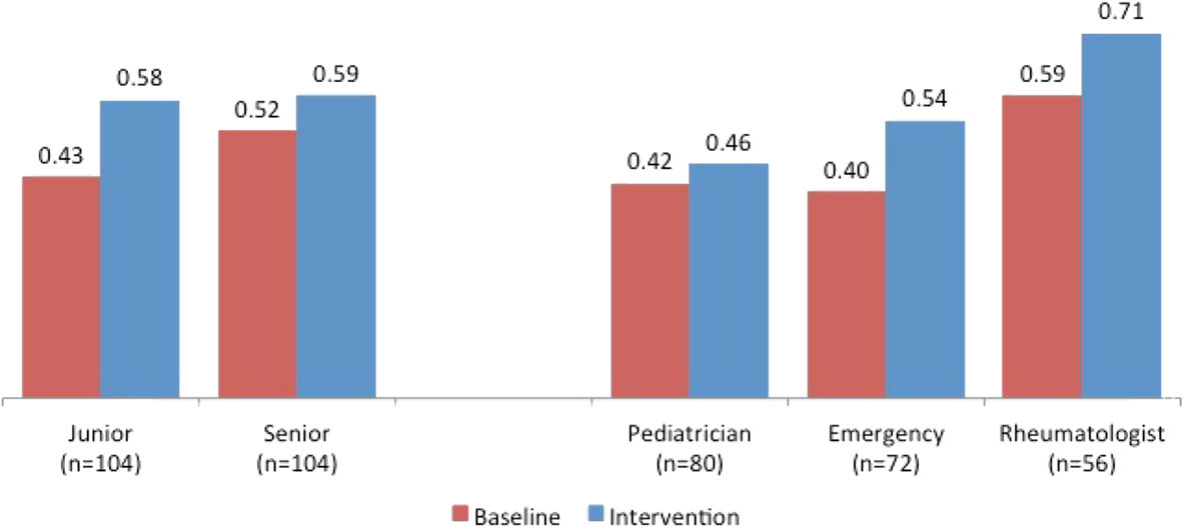
Change in relevance of the diagnosis before and after decision support, by seniority and specialty

This decrease in listed diagnoses was due in part to testers listing fewer irrelevant diagnoses after using the diagnostic support software (Fig. 3). Diagnoses of senior clinicians were more relevant than those of other groups at baseline, with a higher fraction of testers’ diagnoses correlating with the gold standard differential diagnoses, but juniors were able to match seniors after using the decision support. Baseline relevance and aided relevance were highest for the pediatric rheumatologists. Relevance improved for all three groups of specialists. With the assistance of the DDSS, non-rheumatologists approached the baseline performance of rheumatologists, but did not match it, as assessed by diagnostic error and relevance (Fig. 3, right).

Testers spent an average of 20 min per case (Table 3), of which half was spent using the decision support. Use of other reference sources while developing the baseline differential diagnosis consumed an average of 8.6 min.

**Table 3:** Time spent using other resources and decision support by case (mean in minutes)

Case	Case summary	Before decision support	While using decision support
1184	*16 year-old boy with multiple joint pains*	14.5	13.8
2088	*17 year-old woman with rash, joint pain and swelling*	7.8	10.9
3613	*18 year-old woman with joint pain and fever*	7.6	12.1
4967	*8-year-old girl with rash and tenosynovitis*	5.0	7.8
5615	*10 year-old girl with elbow pain*	6.5	8.9
6295	*8 year old girl with rash, fevers and joint pain*	9.7	11.5
7870	*11 year-old boy with joint pain and fever*	6.7	9.5
8434	*18 year-old girl with joint pain, rash and fatigue*	6.8	10.1
	Average	8.6	10.9

### Analysis of diagnostic errors

In order to identify potential ways to improve the diagnostic support software, we made use of the Baseline versus Intervention information for each testing instance to examine cases in which testers changed to or from a diagnostic error. In 33 instances, the tester changed from error (diagnosis or category not in the gold standard list of diagnoses) to correct response after using the software (Table 4; “Fixed errors”). In seven instances, the tester changed from correct to error (“Added errors”). In other instances, no change was made. Overall, improvements were made in 83% of instances in which changes were made, including 100% of changes made by rheumatologists and by junior emergency medicine physicians. Changes in diagnoses led to improvements in 91% of changes made by junior clinicians, while senior clinicians improved in 72% of changes.

**Table 4:** Changes by type of tester

	Baseline errors	Fixed errors	Added errors	Percent of changes that reduced errors
ALL				
Rheumatologist	9	5	0	100%
Emergency	26	18	2	90%
Pediatrics	23	10	5	67%
Total	58	33	7	83%
JUNIORS				
Rheumatologist	5	4	0	100%
Emergency	13	10	0	100%
Pediatrics	8	6	2	75%
Total	26	20	2	91%
SENIORS				
Rheumatologist	4	1	0	100%
Emergency	13	8	2	80%
Pediatrics	15	4	3	57%
Total	32	13	5	72%

The state of the software was recorded in each testing instance, allowing analysis of findings input by the testers and diagnoses displayed. In the seven testing instances with new errors, there were a variety of input errors, including findings in the case entered without onset ages, findings omitted entirely or interpreted incorrectly; findings not representing information in the vignette being entered; or diagnoses high in the software’s differential diagnosis rejected by the tester.

### Results varied by tester

**Fig. 4:**
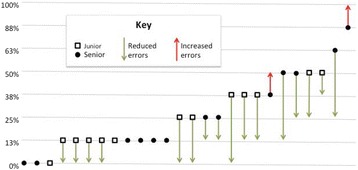
Net diagnostic errors by tester

For the 26 testers, baseline diagnostic errors clustered around the mid-values (17/26 testers with 13%–38% errors; Fig. 4). After using diagnostic software, diagnostic errors clustered around a lower error rate (20/26 with 0%–13% errors). Two of the testers had a net increase in errors (Fig. 4). These two testers out of 26, both senior pediatricians, accounted for 29% of new errors (2 of 7), and 40% of unchanged errors (10 of 25). None of their changes led to improvements (0 of 2), whereas the remaining 24 testers had 85% of changes leading to improvement (33 of 38).

### Results varied by case (Additional file 1)

In seven of eight cases, there was a reduction in overall diagnostic errors after use of decision support. In one case there was no change in total (Table 5). In six of eight cases the average baseline error before decision support was 15%, but in two cases the error rates before decision support were 58 and 73%. The overall likelihood of improvement in situations in which the tester made a change was 83%, however, in the two cases with the most baseline errors, 95% of changes resulted in improvement.

**Table 5:** Changes by case (*n* = 26 for each case)

Case	Baseline errors	Fixed errors	Added errors	Percent of changes that reduce errors
1184	19	8	1	89%
6295	15	10	-	100%
3613	8	3	2	60%
2088	4	3	-	100%
8434	4	3	-	100%
7870	3	2	1	67%
5615	3	3	2	60%
4967	2	1	1	50%
TOTAL	58	33	7	83%

Similar analyses were done for workup lists, but no significant changes were found.

## Discussion

This study examined the effect of diagnostic decision support starting from a baseline of a clinician’s knowledge and unlimited availability of information resources such as books and web searches. The testers spent almost as much time with outside medical resources as they did using the decision support software (8.6 vs. 10.9 min), but the improvement in diagnostic accuracy attributable to the software was nonetheless highly significant.

In actual clinical practice, if generalists were able to lower their diagnostic errors to such an extent, we would expect that the utility of subspecialty referrals would improve significantly. For example, a review of referrals to the rheumatology clinic at Boston Children’s Hospital suggests that at least 10% could have been avoided or directed to more appropriate consultants with minimal diagnostic support (unpublished data). Such a reduction in sub-optimal referrals would meaningfully reduce unnecessary capacity utilization and shorten wait times for the limited number of available clinic appointments.

In this study, junior rheumatologists equipped with decision support were able to rival the diagnostic accuracy of their unaided senior colleagues, an improvement that could further extend the availability of pediatric rheumatology consultations. However, not all clinicians appeared to benefit, with two of the 26 testers, both senior pediatricians, having none of their changes after diagnostic software leading to improvements, versus 85% of changes leading to improvements for the other 24. Better understanding of the reasons that benefits of using the software varied so widely between individuals will be necessary to optimize the value of DDSS.

While medical decision support systems have been anticipated since the earliest years of computers, meaningful attempts to incorporate such tools in the practice of medicine largely have been unsuccessful. Even as computational power and software capabilities have increased exponentially, progress in machine-assisted diagnosis has been far less impressive [26]. This is often ascribed to difficulty developing heuristics of the human diagnostic process [27]. In an attempt to overcome these challenges, SimulConsult® diagnostic software incorporates such components as temporal evolution of signs, symptoms and diseases and use of pertinent negatives to improve diagnostic decision making. It already forms the basis of widely used support software adopted as a primary educational tool by the Child Neurology Society and mentioned by the UK Health Departments as a means of facilitating rapid diagnosis of rare diseases [28].

Rheumatology, however, poses different challenges than previous areas in which SimulConsult® has been proven useful (neurology, genetics and genome-phenome analysis). For example, rheumatologic diagnoses rest primarily on precise recognition of physical findings, rather than confirmatory serologic, genetic or imaging findings. In order to adapt the strengths of SimulConsult® to these aspects of rheumatologic decision making, the present iteration of this software included an explicit review of the curation using a rheumatology-specific set of core clinical findings. While many rheumatologic and non-rheumatologic findings were used in curation of information for diagnostic assistance, this study added a new step in which a core set of 46 rheumatologic findings was explicitly reviewed in each rheumatologic disease. Such an approach was recommended in our previous study because the same set of findings could also be used for initial entry of patient information [23]. This study suggests that this approach is particularly practical in rheumatology due to the relatively small number of clinical findings, compared with the much larger numbers in other areas such as dysmorphology [29]. However, the crucial importance of correct recognition and recording of physical exam and historical findings was beyond the scope of this study. Rather than vignettes as were used in this study, answering this question will require actual patients being assessed in real time. Such an approach will present its own challenges, including metrics to assess the software’s utility when each case will be amenable to only a single evaluation.

A similar curation of core rheumatologic laboratory or imaging tests will be central to the next version of the rheumatologic DDSS. In particular, it will be important to specify findings on laboratory tests and imaging studies in rheumatologic conditions as thoroughly as rheumatologic diagnoses are now curated. This will both improve the ability of the software to help optimize the diagnostic work-up, as well as allow for quantification of potential cost-savings attributable to more efficient diagnosis. Another need identified was to group clinical tests, such as measurements of proteinuria and hematuria in a urinalysis “bundle”. This approach was shown previously to be important as a means of more closely approximating the clinical utility of radiologic tests [23]. In addition, as noted in the previous study, while diagnosis generally has an objectively confirmed correct answer, there is often far less agreement on prioritization of studies during the diagnostic workup, with such vagaries evident even in guidelines proposed by national governing bodies [30].

Even more than in neurologic and genetic disorders, the time course of evolution of clinical findings plays a major part in generating a differential diagnosis in pediatric rheumatology. Most of the diseases evolve over several weeks to years, as for example, scleroderma. However, others evolve over a period of hours to days, such as Henoch–Schönlein purpura, Kawasaki disease and macrophage activation syndrome. Therefore, in carrying out this study we added a new type of finding to represent acuity of onset of diseases. Although the tool has a detailed model of onset ages and disappearance ages for individual findings in each disease, there had previously been no way of representing whether the clinical manifestations of a disease emerge over minutes, days or years. In this study, curation of such acuity information was done for many conditions, but it was not applied systematically to all possible diagnoses. Consequently, although such acuity often did appear among the findings suggested as useful, they could not be applied routinely to diagnostic assessments since relevant data were often missing, particularly for non-rheumatologic mimics of rheumatologic conditions. Additional systematic curation of the range of onset acuities will improve diagnostic accuracy, and augment the utility of the software in settings in which temporal information is particularly important, such as emergency departments and intensive care units.

One of the questions assessed in this study was whether separate, disease-specific diagnostic tools are necessary for different specialties. Eventually, in order to be useful in emergency departments and primary care clinics, the software will have to be able to distinguish joint pain and swelling due to juvenile arthritis, mucopolysaccaridoses, leukemia and Lyme disease. Part of the rationale of choosing rheumatology for this study was its perceived low overlap with genetics and neurology information already in the tool, allowing assessment of whether combining these different areas was feasible. During this study it became clear that the overlap with areas such as neuromuscular neurology was greater than anticipated. This adds to our assessment that separating coverage of these diseases into different diagnostic tools might well be disadvantageous, potentially contributing to balkanization of information and increasing diagnostic errors due to “premature closure.” This became apparent early in the study when the tool was demonstrated to the pediatric rheumatology program at Boston Children’s Hospital. A case that had been perceived as diagnostically difficult within the department because the diagnosis was outside of rheumatology actually converged rapidly to a diagnosis once the findings were entered into the diagnostic software tool.

In summary, our data suggest that SimulConsult® diagnostic software can be most useful in two situations: 1. To help rheumatologists consider disorders typically outside of rheumatology and 2. To help non-rheumatologists generate better-informed and timely referrals, thus improving utilization of scarce rheumatology expertise. Since we saw examples of each of these phenomena in the process of planning and carrying out this study, and the results of the study confirmed significant diagnostic benefit from the software for both rheumatologists and non-rheumatologists, we conclude that the approach of integrating diagnostic decision support for rheumatology into clinicians’ workflow is practical and promising. It seems likely that a single diagnostic support platform may encompass many more medical specialties. One of the initial benefits of such an approach will likely be greater access to diagnostic expertise for underserved specialties such as pediatric rheumatology,

In practice, such generalized diagnostic tools would offer maximal utility for helping direct patients to appropriate specialists and appropriate centers of excellence. In contrast, the alternative approach of having separate diagnostic tools for different areas would only exacerbate the tendency of organ system specific specialties to function within non-communicating universes [31]. Although extra effort will be necessary to construct tools that integrate many disciplines, the advantages clearly seem to warrant such an undertaking.

## Conclusions

We assessed generalists’ and specialists’ ability to accurately diagnose patients presenting with rheumatologic signs and symptoms using actual case summaries. Testers were then allowed to reconsider their diagnoses using the SimulConsult® tool. This diagnostic decision software was originally created for use in neurology and genetics, and for this study had been augmented with information concerning rheumatologic conditions in childhood. Diagnostic errors were decreased by 45% overall, and among emergency medicine physicians by 62%. Pediatric rheumatologists’ ability to diagnose non-rheumatologic mimics of rheumatologic diseases, such as metabolic disorders, appeared to be improved as well. We conclude that diagnostic decision software utilizing novel pattern matching, taking into account the temporal pattern of clinical findings, and incorporating a multidisciplinary database holds promise as a means of improving diagnosis of patients with rare diseases, particularly in underserved specialties such as pediatric rheumatology.

## Additional file

Additional file 1: Vignettes supplementary material. (DOCX 21 kb)

## Abbreviations

ANA: Anti-nuclear antibody
DDSS: Diagnostic decision support software
ESR: Erythrocyte sedimentation rate
GEE: Generalized estimating equations
NIAMS: National Institute of Arthritis and Musculoskeletal and Skin Diseases
UK: United Kingdom

## References

[1] American Board of Pediatrics. Workforce data. https://www.abp.org/content/workforce-databook. Accessed 1 July 2016.

[2] Dejaco C, Lackner A, Buttgereit F, Matteson EL, Narath M, Sprenger M. Rheumatology work force planning in Western countries: A systematic literature review. Arthritis Care Res. 2016. Accepted Author Manuscript. doi: 10.1002/acr.22894 10.1002/acr.22894 27015436

[3] Arthritis Foundation. Pediatric Rheumatologist Map 2015. at http://www.arthritis.org/images/sections/advocate/Pediatric-Rheuma-Map-8.5x11-2015-vf.png. Accessed 31 July 2016.

[4] Mayer ML (2006). Are we there yet? distance to care and relative supply among pediatric medical subspecialties. Pediatrics.

[5] Al Maini M, Adelowo F, Al Saleh J (2015). Clin Rheumatol.

[6] Kumar, Bharat. Global health inequities in rheumatology Addressing an urgent and largely unmet need of the developing world. Rheumatol. 2016. http://rheumatology.oxfordjournals.org/content/early/2016/03/24/rheumatology.kew064.full.pdf+html.

[7] Henrickson M, Henrickson M (2011). Policy challenges for the pediatric rheumatology workforce: part III. The international situation. Pediatr Rheumatol Online J.

[8] Oyoo O, Moots RJ, Ganda B. "Stepping into the state of rheumatology in East Africa." Rheumatology (2012): ker411. http://rheumatology.oxfordjournals.org/content/51/8/1345.full.pdf+html. 10.1093/rheumatology/ker411 22319079

[9] McGhee JL, Burks FN, Sheckels JL, Jarvis JN (2002). Identifying children with chronic arthritis based on chief complaints: absence of predictive value for musculoskeletal pain as an indicator of rheumatic disease in children. Pediatrics.

[10] Correll CK, Spector LG, Zhang L (2015). Barriers and alternatives to pediatric rheumatology referrals: survey of general pediatricians in the United States. Pediatr Rheumatol.

[11] Woo P (2009). Theoretical and practical basis for early aggressive therapy in paediatric autoimmune disorders. Curr Opin Rheumatol.

[12] Muhammad Haroon, Phil Gallagher, Oliver FitzGerald. Diagnostic delay of more than 6 months contributes to poor radiographic and functional outcome in psoriatic arthritis. Ann Rheum Dis doi:10.1136/annrheumdis-2013-204858. 10.1136/annrheumdis-2013-204858 24525911

[13] Minich LL, Sleeper LA, Atz AM (2007). Delayed diagnosis of Kawasaki disease: what are the risk factors?. Pediatrics.

[14] Henrickson M (2011). Policy challenges for the pediatric rheumatology workforce: part II. Health care system delivery and workforce supply. Pediatric Rheumatol.

[15] Deal CL, Hooker R, Harrington T (2007). The United States rheumatology workforce: supply and demand, 2005–2025. Arthritis Rheum.

[16] Marcin JP, Rimsza ME, Moskowitz WB, Committee on Pediatric Workforce (2015). The use of telemedicine to address access and physician workforce shortages. Pediatrics.

[17] Feldman L (2009). Managing the cost of diagnosis. Manag Care.

[18] Schiff GD, Hasan O, Kim S (2009). Diagnostic error in medicine. Analysis of 583 physician-reported errors. Arch Intern Med.

[19] Newgard CD, Zive D, Jui J (2013). Electronic versus manual data processing: evaluating the use of electronic health records in Out-of-hospital clinical research. Acad Emerg Med.

[20] Segal MM, Abdellateef M, El-Hattab AW (2015). Clinical pertinence metric enables hypothesis-independent genome-phenome analysis for neurological diagnosis. J Child Neurol.

[21] Berner ES, Webster GD, Shugerman AA (1994). Performance of four computer-based diagnostic systems. N Engl J Med.

[22] Ramnarayan P, Kapoor RR, Coren M (2003). Measuring the impact of diagnostic decision support on the quality of clinical decision making: development of a reliable and valid composite score. J Am Med Inform Assoc.

[23] Segal MM, Williams MS, Gropman AL (2014). Evidence-based decision support for neurological diagnosis reduces errors and unnecessary workup. J Child Neurol.

[24] Segal MM. Systems and methods for diagnosing medical conditions. Methods described in US Patent 6,754,655. Issued June 22, 2004.

[25] Vittinghoff E, Glidden DV, Shiboski SC, McCulloch CE (2005). Regression methods in biostatistics: linear, logistic, survival, and repeated measures models.

[26] Bond WF, Schwartz LM, Weaver KR (2012). Differential diagnosis generators: an evaluation of currently available computer programs. J Gen Intern Med.

[27] Wegwarth O, Gaissmaier W, Gigerenzer G (2009). Med Educ.

[28] Consultation on the United Kingdom Plan for Rare Diseases 2012; https://www.gov.uk/government/uploads/system/uploads/attachment_data/file/215141/dh_132883.pdf.

[29] Allanson JE, Biesecker LG, Carey JC, Hennekam RC (2009). Elements of morphology: introduction. Am J Med Genet A.

[30] Tozzoli R, Bizzaro N, Tonutti E (2002). Guidelines for the laboratory use of autoantibody tests in the diagnosis and monitoring of autoimmune rheumatic diseases. Am J Clin Pathol.

[31] Hanauef SB (2010). A poor view from specialty silos. Nat Rev Gastroenterol Hepatol.

