# Supplementary material for: Evidence-based decision support for pediatric rheumatology reduces diagnostic errors
Source: Pediatr Rheumatol Online J. 2016 Dec 13;14:67. doi: 10.1186/s12969-016-0127-z (PMC5155385; doi:10.1186/s12969-016-0127-z)
Supplement: Supplementary file 1 — Vignettes supplementary material. (DOCX 21 kb) [file 12969_2016_127_MOESM1_ESM.docx]

### Case 1184:

### 16 year-old boy with multiple arthalgias

A 16-yo boy comes to his pediatrician complaining of diffuse extremity and joint pain. He had been seen in the office previously for intermittent episodes of pain in his hands, wrists, feet and ankles accompanied by fever. He was first seen by a rheumatologist for these complaints when he was 13, but full physical exam failed to confirm the presence of joint inflammation, and laboratory studies, including genetic testing for periodic fever syndromes, were negative. He was treated for presumed atypical systemic Juvenile Idiopathic Arthritis with nonsteroidal antiinflammatory drugs and prednisone without benefit.

The episodes have continued and gradually worsened, but lately his joints have become more painful and stiff and his extremity pain has become more constant. He has also developed a rash on his flanks. Review of systems is notable for diarrhea that does not resolve with avoidance of dairy products or other dietary manipulations. He also reports foamy urine and poor exercise tolerance, often having to drop out of pick-up basketball games because of pain and burning of his palms and soles. This pain improves when he stops playing, but never resolves completely. He has had no fevers and he sleeps well, keeping hours like those of many of his high school classmates and generally not napping. Family history is notable for a mother who died of congestive heart failure at the age of 63 and a grandmother who died of chronic renal failure at the age of 66 after three years of hemodialysis.

On exam, the young man is afebrile and has a normal blood pressure. He has limited flexion of the fingers, wrists and ankles, but no swelling, warmth or tenderness of any joint. There is atrophy of the intrinsic muscles of the hands and increased sensitivity of palms to even gentle touch. He has a non-blanching rash on his lower abdomen and flanks that is minimally tender to palpation. The remainder of his physical exam is unremarkable including no murmurs, rubs or gallops, clear breath sounds, benign abdomen without organomegaly.

### Case 2088:

### ****17 year old woman with rash and joint pain and swelling****

A 17 yo woman presented to the Emergency Department at 3 AM because severe right wrist pain. The pain had started the previous evening and had worsened despite use of ibuprofen 600 mg every 4-6 hours. She left work early that afternoon when she developed fever, went to bed early, but awoke at midnight with unrelenting pain. She therefore came to the emergency department. Review of systems is negative for nausea, vomiting, diarrhea, bloody stool, swollen glands, photophobia or ocular irritation. Past medical and family history are unremarkable.

On physical examination she is febrile to 101° F, with a heart rate of 88 beats per minute and a normal blood pressure. Skin was notable for tender, non-blanching pustules on both palms. Her right wrist was swollen, warm and exquisitely tender to palpation with swelling extending over the dorsal surface of the hand. Even the slightest movement in any plane was resisted. Other joints had full range of motion without evidence of inflammation. The remainder of her physical exam was entirely normal.

### Case 3613:

### ****18 year old woman with joint pain and fever****

An 18 year old young lady presents to her primary caregiver with daily fevers to 101° F, a faint erythematous rash, and wrist and knee pain. He diagnoses her with a viral syndrome and gives her ibuprofen 600 mg tablets which she takes 2-3 times per day with minimal improvement. Over the next two weeks symptoms persist and pain spreads to both ankles and the left shoulder, and she develops chest pain that is worst when she goes to bed. She returns to her pediatrician. Review of systems is notable for a four pound weight loss since symptoms began almost three weeks ago. Past medical history is notable for an episode of knee pain and fever improving with a 6-days tapering course of prednisone two years earlier.

On exam she is tired appearing with pale conjunctivae, mild tachycardia to 105 beats per minute and normal blood pressure. She has a blanching, erythematous maculopapular rash on her abdomen, thighs and back. There are several 1-2 cm anterior cervical and axillary lymph nodes. Heart sounds are slightly muffled, and the patient has Grade 2 holosystolic murmur at the left sternal border, though the patient is unable to lean forward during the exam for more than a minute without developing chest pain. Musculoskeletal exam reveals swelling, warmth and tenderness of both knees, wrists and left shoulder. Range of motion in involved joints is limited due to pain.

An X-ray of the right wrist revealed erosions at the proximal 2nd and 3rd metacarpal bones.

| Case 4967: |
| --- |

**8-year old girl with rash and tenosynovitis**

The girl was in her usual state of health until she developed erythematous patches on her elbows. This was treated with topical emollients without benefit. One month later she awoke with pain and stiffness of her heel and ankle, and was brought to see her pediatrician. Trauma during a weekend soccer game was suspected and she was given an ACE bandage and referred to physical therapy. At  physical therapy, swelling of the girl’s left ankle was noted and the family was asked to see a rheumatologist.

When seen by the pediatric rheumatologist four weeks after her foot symptoms began, the patient reported pain upon arising in the morning which lasted until early afternoon. She had to stop playing soccer because of heel pain. Her rash was also waxing and waning, tending to worsen on cold and dry days. On physical exam, she was a well-appearing, well-nourished girl in no acute distress. She had a scaling rash over the extensor surfaces of both elbows and at the medial canthus of her left eye. Both ankles were swollen, tender and  warm, with decreased extension and inversion. Achilles tendons were swollen with tenderness at the insertions into the calcanei bilaterally. The right index finger had diffuse swelling and tenderness of the proximal interphalangeal joint, with sausage-shaped fullness and tenderness extending to the middle of the proximal and distal phalanges. Flexion of the index finger was limited. The remainder of the exam was normal.

| Case 5615 |
| --- |

**10 year old girl with elbow pain**

The patient was brought to her pediatrician with a painful left elbow suffered when she hyperextended the joint while playing with friends. She was prescribed ibuprofen 400 mg twice daily, and her left arm was placed in a sling for two weeks. When the sling was removed she felt better, though she continued to have intermittent pain localized to the region of the left epicondyle as well as occasional stiffness, treated with splinting as needed.

The following autumn she suffered a fall to her right shoulder during dance practice. She had some difficulty with right shoulder movement initially, improving with physical therapy though continuing to experience intermittent pain. Three months later she developed hip pain while dancing and was referred to orthopedics. She underwent physical therapy with some improvement though she had to stop dancing because of ongoing pain. She was referred to rheumatology with a question of arthritis.

The patient’s review of systems includes intermittent pain involving her left elbow, right shoulder and bilateral hips. She also describes TMJ discomfort with chewing as well as occasional "clicking" sounds. She greatly misses being able to dance, but otherwise reports good health. Family history is notable for Lilly’s mother and maternal aunt with vertebral disc problems in their early 20's. Physical exam shows no swelling, erythema or warmth of any joints, though Lilly’s wrists, shoulders, hips and temporomandibular joints are mildly tender to palpation. Lilly likes to demonstrate some of her dance moves, including placing her palms on the floor when she bends at the waist while knees are extended. Her exam and laboratory studies are otherwise normal.

| Case 6295 |
| --- |

**8 year old girl with rash, fevers and arthralgias**

The patient is an 8 year-old girl who began to have pain in both elbows, both knees and neck one week ago. The next day she developed daily fevers up to 102° F with fatigue and malaise, followed shortly by development of a diffuse, slightly raised and occasionally pruritic rash. Treatment with acetaminophen resulted in only transient improvement.

She was seen by her pediatrician two days ago when she developed severe abdominal pain after eating. She was referred to the Emergency Room where she was switched to round-the-clock ibuprofen and referred to rheumatology.

The patient’s past medical history is notable for a strep throat approximately 6 weeks ago that was successfully treated with penicillin for 10 days. On review of systems she reports development of headaches approximately coincident with the onset of her arthralgias. She denies associated photophobia or phonophobia. She does note the rash on her extremities, especially the feet, treated with diphenhydramine for the itch. She denies any vomiting or diarrhea but her appetite has decreased significantly since she developed severe post-prandial abdominal pain. She denies mouth sores or dysphagia; visual changes, shortness of breath or chest pain. She denies Raynaud’s symptoms or conjunctivitis.

Her only medications are Ventolin and Cromolyn metered dose inhalers as needed for bronchospasm which is usually triggered by exercise and upper respiratory infections. She has some seasonal allergies but no known drug allergies. Family history is noncontributory.

Physical examination is notable for T=102.8o F, blood pressure of 146/98 (144/102 on repeat). Her weight is 80th %ile and her height 50th %ile. She is well-appearing, slightly pale, with a serpiginous livedoid rash on her extremities, legs more than arms. Her neck is supple with full range of motion, some tenderness at the posterior sternocleidomastoid muscles, no palpable lymphadenopathy. Extremities are warm and well-perfused without cyanosis, clubbing or edema. Both knees and both elbows have moderate effusions, and flexion is splinted secondary to pain. She has 0.5-1 cm tender, erythematous nodules over her distal wrists and fingers. No other joint has evidence of inflammation, and all other joints have full range of motion. Neurologic examination is non-focal and symmetric with 5/5 strength, normal coordination and 2+ reflexes throughout with downgoing toes bilaterally. Pulses are bilaterally symmetric and normal. Allen test is notable for delayed filling of the left hand when pressure is applied to the radial artery. No bruits are appreciated.

| Case 7870 |
| --- |

**11 year old boy with joint pain and fever**

An 11 year old boy presented to the emergency department with pain in his right hip. For the previous four days he had complained of headache and sore throat, and his mother had thought that his forehead felt warm. In the ED he had a temperature of 100.4° F, several 1-2 cm anterior and posterior cervical lymph nodes, and a scanty exudate on his tongue and tonsils. He resisted internal rotation and full extension of his right hip, but other joints were normal.  A hip ultrasound showed an effusion and orthopedics aspirated 3 ml of clear fluid with a cell count of 1450/mm^3^. He felt better and was diagnosed with toxic synovitis and told to use ibuprofen as needed until symptoms resolved.

The boy returned to school two days later and was completely well for several days apart from half a day of right wrist pain that improved with ibuprofen. The next day, however, he had to sit out of gym class when he became short of breath while playing basketball, and later in the day he developed bilateral ankle pain and swelling despite taking ibuprofen. He was brought back to the emergency room. On examination he was uncomfortable and had a temperature of 100.7° F, heart regular at a rate of 128, and respiratory rate of 34. He had an erythematous serpiginous rash over his trunk and upper thighs. Cardiac exam was notable for tachycardia and a grade 2/6 basal ejection systolic murmur. Lungs were bilaterally full and clear to auscultation. Abdomen was notable for mild right upper quadrant tenderness to palpation, with the liver edge 7 cm below the right costal margin. No masses or bruits were appreciated. Musculoskeletal exam revealed bilateral swelling, warmth and tenderness of both ankles, decreased range of motion with mild swelling and tenderness in his left wrist and right knee. Both hips were non-tender with full range of motion.

| Case 8434 |
| --- |

**18 year old girl with joint pain, rash and fatigue**

The patient was in her usual state of good health until approximately two months ago, when she developed a blistering sunburn after spending a day at the beach with friends. The sunburn resolved over the next week, apart from several circular reddish-brown crusted lesions on her ears, forehead, and cheeks. Her ankles and knees became painful and stiff, most noticeably in the morning, and opening bottles and jars became increasingly difficult as her fingers and ankles started to swell. She developed daily low-grade temperatures and profound fatigue, needing as much as 14 hours of sleep a day and still falling asleep in class. Her appetite also decreased, and over the past two month she has lost 10 pounds without trying to diet.

The patient was seen by her primary care provider and lab tests were obtained.  She was given naproxen for her joint complaints, and this helped somewhat. However her limitations worsened, and for the past week she has been unable to walk even 100 feet due to the pain and swelling in her ankles and knees. She has also noted excess hair in the bathtub after taking a shower, and the hair around her temples has thinned noticeably. Today the patient’s lab tests returned, and her PCP called to set up an urgent rheumatology clinic appointment.

On review of systems, the patient notes cold sensitivity since the 9th grade, when her fingers started turning dark purple whenever she went outside in the winter without gloves. Socially, after having been an honors student, the young lady is now barely getting C’s on tests, and she is finding herself having trouble concentrating during class.

On physical exam she is tired appearing but in no distress. She has a temperature of 99.5° F and her vital signs are otherwise normal. She has about half-a-dozen quarter-sized purplish-brown lesions on her ears, cheeks, and forehead, all slightly tender to touch. She has shallow off-white 1-2 mm non-tender ulcerations on her palate and bilateral 1-2 cm lymph nodes in the anterior cervical chains bilaterally. Musculoskeletal exam is notable for tender swelling and warmth of her MCP, PIP, wrist, ankle and knee joints, and diffuse tenderness of the muscles of her thighs, hamstrings, holders and forearms. Her ability to flex her wrists, elbows, hips and knees is significantly limited.
